# Toward Making Inroads in Reducing the Disparity of Lung Health in Australian Indigenous and New Zealand Māori Children

**DOI:** 10.3389/fped.2015.00009

**Published:** 2015-02-13

**Authors:** Anne B. Chang, Robyn L. Marsh, John W. Upham, Lucas R. Hoffman, Heidi Smith-Vaughan, Deborah Holt, Maree Toombs, Catherine Byrnes, Stephanie T. Yerkovich, Paul J. Torzillo, Kerry-Ann F. O’Grady, Keith Grimwood

**Affiliations:** ^1^Child Health Division, Menzies School of Health Research, Charles Darwin University, Darwin, NT, Australia; ^2^Queensland Children’s Medical Research Institute, Queensland University of Technology, Brisbane, QLD, Australia; ^3^Department of Respiratory Medicine, Princess Alexandra Hospital, Brisbane, QLD, Australia; ^4^School of Medicine, The University of Queensland, Brisbane, QLD, Australia; ^5^Department of Pediatrics, University of Washington, Seattle, WA, USA; ^6^Department of Microbiology, University of Washington, Seattle, WA, USA; ^7^Indigenous Health, Toowoomba Rural Clinical School, The University of Queensland, Toowoomba, QLD, Australia; ^8^Paediatric Department, University of Auckland & Starship Children’s Hospital, Auckland, New Zealand; ^9^Queensland Lung Transplant Service, The Prince Charles Hospital, Chermside, QLD, Australia; ^10^Nganampa Health Council, Alice Springs and Royal Prince Alfred Hospital, The University of Sydney, Sydney, NSW, Australia; ^11^Gold Coast University Hospital, Griffith University, Gold Coast, QLD, Australia

**Keywords:** Indigenous, acute respiratory infections, outcomes, lung health, bronchiectasis, children

## Introduction

Globally, Indigenous populations, which include Aboriginal and Torres Strait islanders in Australia and Māori people in New Zealand (NZ), have poorer health than their non-Indigenous counterparts (1). Indigenous peoples worldwide face substantial challenges in poverty, education, employment, housing, and disconnection from ancestral lands (1). While addressing social determinants of health is a priority, solving clinical issues is equally important. Indeed, ignoring the latter until social issues improve risks further disparity as this may take generations. A systematic overview of interventions addressing social determinants of health found a striking lack of reliable evaluations (2). Where evidence was available, health improvement associated with interventions was modest or uncertain (2). Thus, advances in healthcare remain essential and these require the best evidence available in preventing and managing common illnesses, including respiratory illnesses.

## The Importance of Respiratory Health in Childhood

Relative to its substantial disease burden, lung health receives little attention worldwide compared with other conditions, which attract substantially more media attention and support from research funding bodies. Yet, pneumonia remains the most important cause of mortality and morbidity in young children globally (3, 4). By 2020, chronic obstructive pulmonary disease (COPD) is expected to be the third-ranked cause of mortality in the world (5). Moreover, COPD, thought previously to occur only in smokers, is recognized increasingly in non-smoking individuals (5, 6). Indeed, those with non-smoking-related COPD may have poorer clinical outcomes (higher hospitalizations for COPD and pneumonia) than smoking-related COPD (6). Non-smoking-related COPD is associated with childhood respiratory infections, as is bronchiectasis (7). The latter often goes under-recognized as 29–50% of people with severe COPD (8), and as many as 40% of patients with difficult to control asthma and chronic cough, have bronchiectasis (9).

To reduce chronic pulmonary disease in adults, interventions during critical phases of lung development in early childhood and even in the pre and ante-natal periods may be necessary (10–12). Unlike some other organs, the lungs continue to develop throughout early childhood (13). Furthermore, pulmonary immunity and respiratory phenotype are influenced by gene–environment interactions early in life (including *in utero*) (14). If lung function is already decreased in infancy, this can persist (track) throughout childhood and predict future respiratory morbidity (15). Indeed, there is increasing evidence that much adult lung disease [such as COPD (6) and bronchiectasis] originates in childhood (where it is potentially preventable) (16, 17).

There are few published Australian data on the changes in the epidemiology and burden of lung health in Indigenous children and adults. Between 2001 and 2012, the overall mortality of respiratory-related deaths declined, by up to 32% in adult males, but respiratory disease still accounts for 8% of the health-gap in mortality between Indigenous and non-Indigenous Australians (18). Infant mortality [respiratory illness is second only to congenital conditions as a cause of death in this age group (19)] has also declined in Australia, but chronic respiratory diseases are being identified increasingly (20). Whether this is related to improved case ascertainment or an increased in prevalence is unknown. In NZ, comparable data are unavailable, but acute admissions (which in children are mainly from respiratory infections) for infectious diseases increased between 1989–93 and 2004–08 from 20.5 to 26.6% of all hospitalizations (21). This was accompanied by an increasing disparity in the burden of infectious diseases experienced by Māori where the age-standardized rate ratio for hospitalization was 2.15 (95% CI 2.14–2.16) compared with European and other non-Pacific groups (21).

Currently, in nation-wide data for Indigenous Australians, respiratory disorders are the most common reason for general practice encounters, the second most prevalent self-reported chronic condition and the second most common cause for hospitalization (22). In NZ, mirroring the situation with infectious diseases, respiratory hospitalizations for Māori children are almost twice that for European and other non-Pacific ethnic groups (23). To help reduce the disparity of lung health in Australian and NZ Indigenous children, a partnership involving Indigenous leaders, pediatric and adult clinicians, laboratory scientists, and educationalists was formed in November 2012 under the umbrella of the Centre of Research Excellence (CRE) in Indigenous Children’s Lung Health, funded by the National Health of Medical Research Council (Australia). Our overarching aim is to improve the lung health of children, particularly Indigenous children, through research. Here, we describe our framework and key discussions from our second workshop that brought together partners of our CRE, external experts, and higher degree research scholars involved in projects linked with the CRE, whose emblem is displayed in Figure S1 in Supplementary Material.

## The Conceptual Framework for Developing Severe Bronchiectasis and COPD

**Figure 1:**
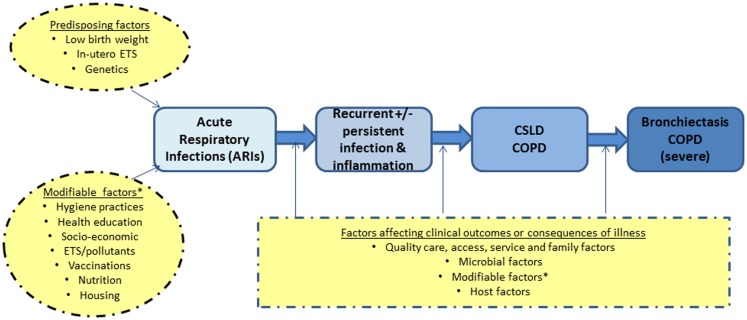
**A simplified diagram on the development of severe lung disease in adulthood (blue) and the possible factors (yellow) that likely influence the progression of disease**. COPD, chronic obstructive pulmonary disease; CSLD, chronic suppurative lung disease; ETS, environmental tobacco smoke exposure.

Our simplified framework for the development and progression of bronchiectasis and/or COPD in adults is presented in Figure 1. The idea that recurrent acute respiratory infections (ARIs) and subsequent bronchiectasis can be prevented is not new (24, 25). In the 1940–50s, astute clinicians described a “pre-bronchiectatic state,” which can lead to irreversible lung disease if not well managed (25). They also recognized that persistent or recurrent lower airway infections were associated with developing bronchiectasis (24).

The alveolar stage of human lung development begins at 36-weeks gestation and continues for ~7 years post-natally (13). Insults in the first few years of life, when post-natal lung development is the most important, are thus likely to have long-term effects. While low birth weight can influence future lung function, there is increasing evidence that early life events (e.g., ARIs) are equally important determinants of adult lung dysfunction, as shown in both animal and human studies (26–29). Therefore, interventions that reduce ARIs in Indigenous children would be expected to have both short and potential long-term benefits, especially as severe and recurrent ARIs are independent risk factors for subsequent COPD (26, 27) and bronchiectasis (7).

Chronic suppurative lung disease (CSLD) in children is a specific clinical phenotype involving symptoms and signs of chronic endobronchial purulence accompanied by persistent or recurrent wet cough and airway inflammation, with or without radiographic evidence of bronchiectasis (30). The limitations of radiographic diagnosis, particularly in children (17, 31), are recognized. In the Northern Territory (NT, Australia), the incidence of CSLD in the first year of life is 118/100,000; (32) while the national incidence of radiographically confirmed bronchiectasis in the NZ population aged <15 years is 3.7/100,000, twice that of cystic fibrosis (33). In Central Australian Aboriginal children (34) and Alaskan Native children (35), CSLD affects one in every 63–68 children (~1500/100,000), yet there are few resources directed at prevention, clinical management, and research. Deaths are uncommon in children (36), although in NZ a 2% mortality rate as a direct result of bronchiectasis was reported from a single tertiary center (37). In contrast, a substantial proportion of Indigenous Australian adults with bronchiectasis die in their fourth decade of life (38).

In adults, the link between COPD and bronchiectasis is increasingly appreciated (39). Those with severe COPD are more likely to have bronchiectasis (40). Furthermore, bronchiectasis in children and adults is often missed or misdiagnosed as asthma (9, 41), as chronic cough in the general population is dismissed frequently as unimportant. We found recently that >20% of children with chronic cough (>4 weeks duration) had seen a doctor >20 times before referral and receiving appropriate treatment (42). Among Indigenous people in Australia, chronic cough is often “normalized,” in contrast to non-Indigenous Australians (43).

Based on the above framework, our approach to achieve improvements in lung health relevant to Indigenous child health focuses on intervention points that are feasible in the clinical context. Our CRE-related projects encompass various aspects relevant to CSLD (Figure 1); most are listed online^1^. Here, we highlight key discussions points from our recent workshop.

## Indigenous Voices and the Paradigm of Research and Care

The approach within our CRE projects is concurrent health education for child caregivers, using culturally sensitive methods, and participation is informed and supported, leading to very high retention rates in randomized controlled trials (RCTs) (44). Projects are accompanied by knowledge translation plans reviewed and endorsed by an Advisory Board (see text footnote 1). An Indigenous Reference Group (IRG) also provides advice before commencing projects. The IRG remain informed throughout the projects and, on its closure, advises on how to provide feedback^2^.

Our projects have also improved community engagement (45). In NZ, whanau (families) of children with bronchiectasis are a driving force leading to Māori community leaders developing an inaugural website for the purpose of providing education and support for all families with children similarly affected. The children themselves are being encouraged to tell their own stories about their health online. It is hoped that this will be a platform, which will continue to improve awareness and generate increased resourcing for this patient group. However, more can always be done. Indigenous leaders of the group challenged us to take further steps, such as examining the role of traditional healers and medicines in our research, adopting an approach to “take your mob with you,” improving the consent process for Indigenous carers, taking into account intergenerational effects and extending our influence beyond lung health. The importance of the work and voices of the four Indigenous higher degree research scholars were highlighted at the workshop.

While a perception remains that Indigenous populations are “over-researched,” in reality there are few RCTs relevant to ethnic disparities in clinical outcomes (46). Indeed, major funding bodies (e.g., The National Institutes of Health, USA) now have policies to ensure that minority groups and women are included in all clinical trials (47). Given the relative paucity of strategies for recruiting and retaining Indigenous people in RCTs (48), McCallum and colleagues’ study, where >97% of Indigenous children were retained in a RCT (44), is a model that should be extended to other studies.

## Host–Pathogen Interaction in Bronchiectasis – The Many Questions

There are many clinical and research gaps relating to the pathogenesis and management of CSLD, including bronchiectasis. This year’s scientific component of the workshop focused largely on the interactions between the lower airways microbiome, host defense mechanisms, and clinical factors relevant to children with CSLD/bronchiectasis.

Few studies have addressed the host–pathogen interaction in children with, or at risk of, CSLD. Pizzutto et al (49) found that children with CSLD have an altered systemic cell-mediated immune response to non-typeable *Haemophilus influenzae* (NTHi) strains *in vitro*. The peripheral blood mononuclear cells of 80 children with CSLD and 51 controls were stimulated with antigens and mitogens and cytokine responses measured (49). The major finding was that those with CSLD produced significantly less interferon-gamma (IFN-γ) in response to NTHi than healthy controls. This deficient IFN-γ response may contribute to increased susceptibility to NTHi infections and to the pathogenesis of CSLD in children. Findings from a larger group of children found that this deficit can be partially corrected by immunization with a licensed pneumococcal-NTHI protein D-conjugate vaccine (50). NTHi is particularly important in CSLD/bronchiectasis (51) and COPD (52) as it is the most common bacteria cultured from the lower airways of children and adults with both diseases.

Whether variants of the genetically diverse NTHi or other pathogens have different virulence strategies in relation to the airway response is unknown. Such data are important for future vaccine development, especially as epidemiologically unrelated NTHi isolates lacking protein D have been detected in Indigenous population (53). Delineating evolutionary changes within the bacterial genome over time and relating this to differential host responses and disease severity remain elusive. Clinical outcome data associating deficits in T-cell function is another research gap. Further, although relating host responses to a single pathogen is useful for understanding the pathogenesis of CSLD and COPD, this is only a small component of the overall host–pathogen interaction. Increasingly, the complexity of the lower airway pathobiology is being understood, such as the contribution of the airway microbiome, biofilms (54), local innate and adaptive immune responses, the influence of antibiotics upon bacterial communities, and the physiological stresses imposed by the lung microenvironment (55).

Data relating to microbiota in human health and disease are increasing exponentially. However, many challenges remain for respiratory studies, such as specimen types and handling (56), sampling the lower airways in young children, and differentiating cause and effect. Longitudinal cohort studies relating microbiota composition to clinical outcomes and inflammation are needed. The role of host tolerance to members of the microbiota within the respiratory tract is also unknown. The relative clinical significance of core versus satellite microbiota, the interaction of the pulmonary microbiome with respect to invading respiratory viruses and oral and gut microbiota, and the roles of these to the pathogenesis of CSLD/bronchiectasis have yet to be studied. Additionally, the concept of mucosal immunization (57) and/or probiotics (58) protecting against ARIs is intriguing.

## Other Studies and the Need for Studies Addressing Prevention

Our current work has an increased focus on primary and secondary/tertiary prevention and treatment strategies, as outlined on our CRE website. Examples include earlier detection of disease through improving follow-up of children post-hospitalization and improving the treatment for bronchiectasis to prevent or to minimize the progression of lung disease. The importance of the latter was acknowledged by our CRE, particularly with tobacco smoking. Once smoking becomes established, it is difficult to discontinue. If individuals avoid smoking during adolescence, it is unlikely that they will take it up later (59), while in contrast the probability of quitting is inversely proportional to the age of initiation (60). Indeed, a home-visit based RCT, using a culturally sensitive approach undertaken by Indigenous workers in Australia and Māori workers in NZ, was unsuccessful in reducing tobacco smoke exposure of Indigenous children when compared to routine care (61).

## Summary

Addressing health issues in childhood is needed to reduce the long-term impairment and social disadvantage in adults. This paradigm is striking for lung health for many reasons, including the impact of respiratory infections and environmental insults upon post-natal lung development, which increase the risk of future lung disease, such as COPD and bronchiectasis. We have briefly presented our framework for the development of severe COPD and/or bronchiectasis (Figure 1) and our model of research and care. In our second annual workshop, we also highlighted several questions concerning the airway microbiome and related host responses in children with CSLD/bronchiectasis.

Through our CRE, we have built partnerships with Indigenous leaders, scientists, and clinicians working toward a common goal of reducing disparity though high-quality science and care. While we are beginning to improve lung health in Indigenous children through several projects addressing various clinically feasible interventions, much work is still to be done in reducing disparity in lung health between Indigenous and non-Indigenous Australian and NZ peoples.

## Author Contributions

AC conceptualized the framework and manuscript, and wrote the first draft. The section on the microbiota and host response was a collection of ideas from the speakers at the workshop. The writing group amended the manuscript and the extended group reviewed it.

## Conflict of Interest Statement

Anne B. Chang has received funding from GlaxoSmithKline for an investigator driven study on the effects of Synflorix^®^ on airway bacteriology. Keith Grimwood has participated on advisory boards to GlaxoSmithKline on pneumococcal conjugate vaccines. All other authors have no conflicts of interest to declare.

## Supplementary Material

The Supplementary Material for this article can be found online at http://www.frontiersin.org/Journal/10.3389/fped.2015.00009/full

## Footnotes

^1^http://www.menzies.edu.au/page/Research/Centres_initiatives_and_projects/Centre_for_Research_Excellence_Lung_health/

^2^http://www.menzies.edu.au/page/About_Us/Board_and_Menzies_committees/Indigenous_Reference_Group/
