# Supplementary figures and images for: Toward Making Inroads in Reducing the Disparity of Lung Health in Australian Indigenous and New Zealand Māori Children
Source: Front Pediatr. 2015 Feb 13;3:9. doi: 10.3389/fped.2015.00009 (PMC4327127; doi:10.3389/fped.2015.00009)

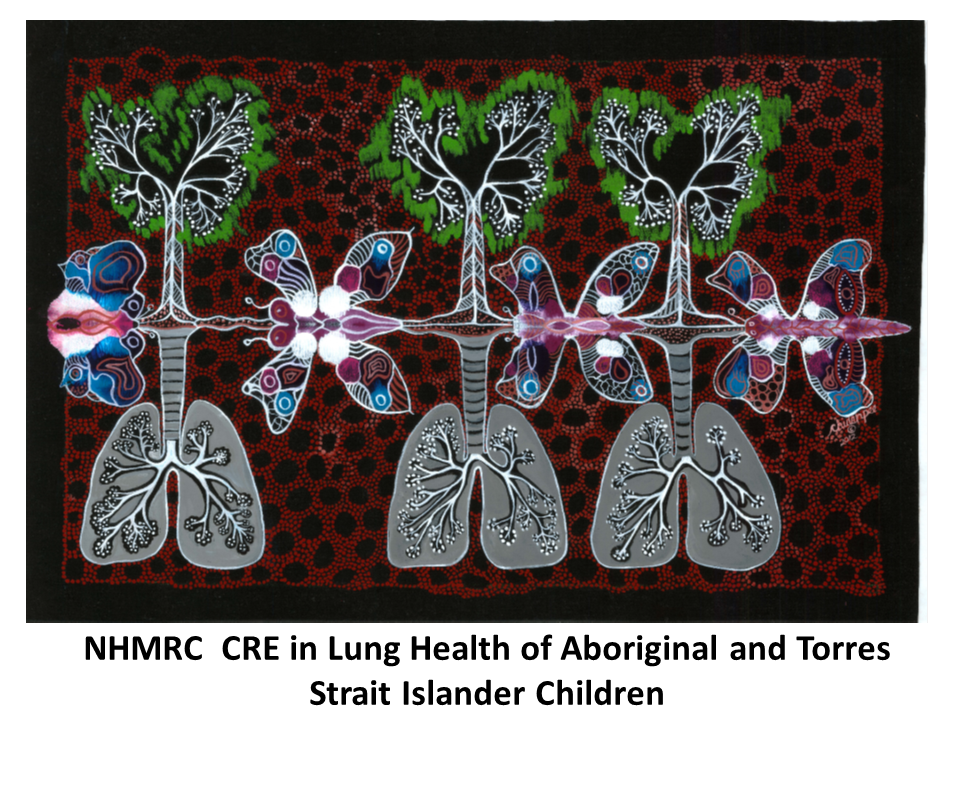

Supplement: Supplementary file 1 [file Image_1.TIF]
